# Clinical findings, synovial fluid cytology and growth factor concentrations after intra-articular use of a platelet-rich product in horses with osteoarthritis

**DOI:** 10.4102/jsava.v90i0.1721

**Published:** 2019-05-23

**Authors:** Yolandi Smit, Hendrik J. Marais, Peter N. Thompson, Arnold T. Mahne, Amelia Goddard

**Affiliations:** 1 Department of Companion Animal Clinical Studies, University of Pretoria, Onderstepoort, South Africa; 2 Saving the Survivors, Pretoria, South Africa; 3 Department of Production Animal Studies, University of Pretoria, Onderstepoort, South Africa; 4 BAKER McVEIGH Cape Town, Milnerton, South Africa

## Abstract

Osteoarthritis is a common cause of lameness in horses, resulting in poor performance. Intra-articular platelet-rich plasma can deliver a collection of bioactive molecules, such as autologous growth factors and proteins involved in the quality of tissue repair. Horses (*n*=5) with osteoarthritis affecting antebrachiocarpal, middle carpal or metacarpophalangeal joints, and horses (*n*=5) without osteoarthritis of the corresponding joints (radiographically free of osteoarthritis), were used for the production of platelet-rich plasma which was subsequently injected into selected joints. Clinical and synovial fluid changes after intra-articular injection of platelet-rich plasma as well as synovial platelet-derived growth factor-BB and transforming growth factor-beta 1 concentration changes were evaluated in these joints and compared between normal joints and joints with osteoarthritis. A gravity filtration system produced a moderately concentrated platelet-rich plasma, representing a 4.7-fold increase in baseline platelet concentration. The synovial effusion score was significantly different between the control joints and joints with osteoarthritis on Day 0 with a higher score in the group with osteoarthritis. Within the control group, the synovial effusion score was significantly higher on Days 1 and 2 compared to Day 0. For both groups, the synovial fluid nucleated cell count, predominantly intact neutrophils, was significantly increased on Days 1 and 2, with no significant difference between groups. The mean synovial platelet-derived growth factor-BB and transforming growth factor-beta 1 concentrations were increased for both groups but significantly lowered in the group with osteoarthritis on Day 1 compared to normal joints. Concentrations for platelet-derived growth factor-BB remained unchanged on Day 5, compared to Day 1, with no significant difference between groups. In conclusion, intra-articular treatment with platelet-rich plasma resulted in increased synovial growth factor concentrations in joints but with lower concentrations in joints with osteoarthritis. A transient inflammatory reaction was seen both clinically as an increase in synovial effusion and cytologically in both normal joints and joints with osteoarthritis.

**Keywords:**

horse; osteoarthritis; platelet-rich plasma; intra-articular.

## Introduction

Osteoarthritis (OA) is one of the most common causes of lameness in horses, resulting in poor performance and economic loss (Sutton et al. 2009). It is a chronic degenerative joint disorder that is multifactorial in origin and characterised by progressive deterioration of articular cartilage, subchondral bone alterations and synovitis (Alcaraz et al. 2010; Mcllwraith, Frisbie & Kawcak 2012). During the development of OA, histological changes include cluster (complex chondrone) formation, fibrillation/fissuring, focal cell loss, increased chondrocyte necrosis and apoptosis, leading to impaired extracellular matrix remodelling (Carmona et al. 2016).

Previously, the pharmacological approach towards OA was confined to symptomatic treatments, such as corticosteroids and hyaluronic acid, which are categorised as symptom-modifying osteoarthritis drugs (SMOADs), aimed at addressing functional impairments and pain severity (Mcllwraith et al. 2012). Over the past decade, regenerative medicine has been considered to be one of the most promising therapies for OA (Filardo et al. 2011; Monteiro, Bettencourt & Lepage 2015). Intra-articular biological therapies that have been described include autologous conditioned serum, stem cells and platelet-rich plasma (PRP) products. These therapies are considered potentially disease-modifying osteoarthritis drugs (DMOADs) (Abellanet & Prades 2009; Fortier 2009; Frisbie et al. 2007; Mcllwraith et al. 2011; Textor & Tablin 2013).

Platelet-rich plasma is defined as an autologous biological product produced from whole blood that contains a higher platelet concentration compared to baseline blood concentrations. The rationale for the use of PRP originates from the fact that not only platelets but also white blood cells (WBC) in PRP contain a wealth of growth factors (Carmona et al. 2016). Platelets contain more than 800 proteins with numerous post-translational modifications for more than 1500 protein-based bioactive factors. Some of these include an important reservoir of growth factors, playing an important role in haemostasis and in processes such as bone remodelling, angiogenesis and healing of damaged tissues. Platelets contain at least 10 growth factors in the alpha-granules, of which platelet-derived growth factor-BB (PDGF-BB) and transforming growth factor-beta 1 (TGF-*β*1) are the most abundant. Upon platelet activation, these growth factors are released from the alpha-granules at the site of injury (Textor & Tablin 2013). Growth factors are biologically active polypeptides with anabolic and anti-catabolic effects that may regulate neo-chondrogenesis, as well as chondrocyte metabolism and differentiation, potentially improving cartilage repair in joints (Fortier et al. 2011; Sun et al. 2010; Van Den Berg et al. 2001; Xie, Zhang & Tuan 2014). Platelet-derived growth factor-BB has been found to downregulate nuclear factor kappa beta (NFk*β*), which is responsible for upregulation of several catabolic and pro-inflammatory cytokines for cartilage (i.e. interleukin-1 beta [IL-1β] and tumour necrosis factor alpha [TNF-α], matrix metalloproteinases, aggrecanases, nitric oxide and cyclooxygenase type 2) and increases chondrocyte proliferation (Carmona et al. 2016). Transforming growth factor-beta 1 contributes to extracellular matrix synthesis and promotes chondrocyte differentiation from stem cells (Carmona et al. 2016). Growth factors, such as PDGF and TGF, are normally low or undetectable in healthy joints (Textor, Willits & Tablin 2013). Textor et al. (2013) found no detectable concentrations of PDGF and very low concentrations (approximately 600–700 pg/mL) of TGF-*β* in healthy joints prior to PRP administration in their study that evaluated growth factor concentrations within synovial fluid pre- and post-PRP administration. According to Van der Kraan (2017), the concentration of TGF-*β* differs greatly between healthy and OA joints, being low in healthy joints and high in OA, leading to the activation of different signalling pathways in joint cells. It has been reported that PDGF has a biological half-life of 2.5 h in synovial fluid after intra-articular PRP administration. However, the release of PDGF is constant, and elevated concentrations can be sustained for at least 7 days. Synovial fluid TGF-*β* concentration can be rapidly induced, and a maximum concentration can be detected within an hour after PRP administration (Roh et al. 2016).

Platelet-rich plasma was first recognised as an effective agent for bone and tissue repair in the field of human dentistry and oral maxillofacial surgery, followed by evidence of improved skin graft wound healing in the field of plastic surgery (Barrientos et al. 2008; Castillo et al. 2011). In recent years, the musculoskeletal effects of PRP have been the main focus in human sports medicine and orthopaedics (Sheth et al. 2012; Xie et al. 2014). The intra-articular use of PRP has been widely reported in human sports medicine, and positive outcomes have been observed in cases of osteochondral lesions and early OA (Filardo et al. 2011; Guadilla et al. 2011; Kon et al. 2011; Mei-Dan et al. 2012; Sampson et al. 2010; Sanchez et al. 2008). Intra-articular PRP has also been used successfully in humans and experimental animals for the treatment of synovitis, cartilage defects, meniscal injury, intra-articular fractures and OA (Ishida et al. 2007; Liu, Yuan & Zhang 2011; Mei-Dan et al. 2012; Saito et al. 2009; Wei et al. 2012).

Serum amyloid A (SAA) is an acute-phase protein (APP) synthesised primarily in the liver that will increase in response to acute inflammation (Jacobsen, Thomsen & Nanni 2006b). In joint disease, the inflammatory mediators present (IL-1*β* and TNF-α) will stimulate the hepatocytes to shift the cellular gene expression towards the production of APPs at the expense of albumin synthesis (systemic acute-phase reaction), resulting in an increase in serum and synovial concentrations of APPs (May, Hooke & Lees 1992). In humans, synovial SAA concentration has been found to be a more sensitive marker of joint disease compared to radiographic examination, and a better indicator of disease activity and prognosis compared to cartilage breakdown products (Jacobsen et al. 2006b). Jacobson et al. (2006b) found an increase in serum and synovial fluid SAA concentrations in horses with experimentally induced OA, but it was of lower magnitude compared to that of infectious arthritis.

Synovial total protein (TP) concentration in OA joints is usually within the reference interval (<20 g/L) or slightly above the upper limit of the reference interval (< 35 g/L) owing to a mild inflammatory reaction present (Steel 2008). The synovial TP concentration may also be affected by various external factors, such as repeated arthrocentesis and intra-articular administration of PRPr (Jacobsen et al. 2006b; Sanchez Teran et al. 2012), that induced a mild acute inflammatory response.

The use of PRP in horses has evolved from the treatment of tendon and ligament lesions to intra-articular therapy, where it is reported to relieve pain and reduce effusion (Carmona et al. 2007; Castelijns et al. 2011). However, studies to assess the response of diseased joints to intra-articular PRP injection, specifically the synovial growth factor concentrations, as well as the changes of SAA and TP in synovial fluid and blood are limited (Carmona et al. 2007; Textor & Tablin 2013; Textor et al. 2013). The objective of this study was to evaluate and compare the changes in clinical signs, synovial fluid cytology, synovial growth factor concentrations (i.e. PDGF-BB and TGF-*β*1), as well as SAA and TP concentrations between normal joints and OA joints after intra-articular injection of PRPr.

## Materials and methods

### Animals

The control group consisted of mature horses (*n* = 5; Nooitgedacht mares) with a mean ± standard deviation (SD) age of 4 ± 0 years with no radiographic and clinical abnormalities of the antebrachiocarpal, middle carpal or metacarpophalangeal joints. The OA group (*n* = 5) (four mares, one gelding, four Thoroughbreds and a Nooitgedacht) with a mean ± SD age of 10.8 ± 6.57 years, had radiographic evidence of OA, affecting the antebrachiocarpal, middle carpal or metacarpophalangeal joints.

One week prior to the study, all the horses underwent lameness, clinical and radiographic examinations. Joints evaluated included antebrachiocarpal, middle carpal or metacarpophalangeal joints. Subjective lameness grades were allocated on a scale of 0 to 5 according to the American Association of Equine Practitioners (AAEP) grading system (0 represented normal gait, and 5 represented non-weight-bearing lameness) (Ross 2011). Joints were clinically evaluated for the response to a flexion test at rest (0-absent, 1-mild, 2-moderate, 3-severe), synovial effusion (0-absent, 1-mild, 2-moderate, 3-severe) and presence of periarticular signs, such as palpable swellings or heat (present or absent). At each time point, days 0 (immediately prior to PRPr intra-articular injection), 1, 2, 5, 21 and 56 post treatment, the lameness and clinical joint examinations were repeated.

Standard radiographic views of the joints were performed as part of the inclusion criteria only. Radiographs were assessed for periarticular osteophytes, joint space narrowing, subchondral bone sclerosis or lysis and/or the presence of osteochondral fragmentation. An OA grade was assigned on a scale of 0–3 with Grade 0: no radiographic abnormalities detected, Grade 1: mild radiographic abnormalities detected (possible osteophytes, doubtful narrowing of joint space), Grade 2: moderate radiographic abnormalities detected (definite osteophytes, some sclerosis/lysis, possible narrowing of joints space) and Grade 3: severe radiographic abnormalities detected (large osteophytes, definite narrowing of joint space, sclerosis/lysis evident, osteochondral fragmentation) to the most severely affected OA joint per horse in the OA group (Frisbie et al. 2007; Holzer et al. 2015; Nganvongpanit et al. 2014).

Horses were excluded from the study if they had intra-articular injections or arthroscopies within 60 days of the study, received medication or oral supplementation for joint disease within 28 days of the study or if there was evidence of a fracture, active infection or a history of chronic infection associated with the joint.

Horses were housed in small paddocks for the duration of the study.

### Preparation of platelet-rich product

An autologous platelet-rich product (PRPr) was prepared using a gravity filtration system, veterinary platelet enhancement therapy (V-PET^™^ [Veterinarian Platelet Enhanced Therapy], Pall Corporation). Fifty-five mL of whole blood was collected from each horse into a 60 mL syringe containing 5 mL of acid-citrate-dextrose A (ACD-A) solution. The anti-coagulated blood was injected into the primary bag containing 9 mL of sterile water. Sterile water was used to promote hypotonic swelling of platelets to assist in their preservation in the filter. After gentle mixing of the solution, the system was suspended with the primary bag at the top, and the solution was allowed to pass through a filter to the secondary bag at the bottom using gravity. Platelets were preserved in the filter and flushed into a sterile syringe, using 5 mL of a 2% saline (NaCl) solution, for the recovery of PRPr. Using sterile techniques throughout, 5 mL of PRPr was retrieved from each horse. One mL of PRPr was retained to determine cellular content, and 4 mL of PRPr was used for the intra-articular treatment.

### Treatment and sampling

Data were collected on days 0 (immediately prior to PRPr intra-articular injection), 1, 2, 5, 21 and 56 post treatment. At each time point, blood was collected by jugular venipuncture into ethylenediaminetetraacetic acid (EDTA) and serum vacutainer tubes for complete blood count (CBC), TP and SAA concentrations. The blood serum sample was left to clot and then centrifuged at 2100 *g* for 8 min and aliquoted into cryovials for storage at -80 °C. On Day 0, blood was collected by jugular venipuncture for PRPr preparation. A 1-mL aliquot of the PRPr was transferred to a standard EDTA tube for analysis.

Arthrocentesis was performed at each time point after sedation of horses with romifidine (Sedivet 1%, Boehringer Ingelheim) (0.02–0.08 mg/kg body weight (BWT) intravenously) as required. Arthrocentesis sites were aseptically prepared, and each selected joint was sampled using a sterile technique. On Day 0, 2 mL of synovial fluid was aspirated from each joint using a 20-gauge, 1.5-inch needle, and 5 mL syringe prior to injection of 4 mL PRPr. For all subsequent sampling days post injection, 2 mL of synovial fluid was collected. Synovial fluid was immediately transferred to EDTA and serum vacutainer tubes for cytological assessment, as well as determination of SAA and TP concentrations. On Days 1 and 5, 0.5 mL of synovial fluid was retained for synovial growth factor concentration analysis. A light bandage was applied to the joint after arthrocentesis.

### Sample handling and analysis

All samples were analysed within 2 hours of collection on an automated analyser (ADVIA 2120, Siemens, Munich, Germany). Data recorded included the platelet count, WBC count and differential leukocyte count, which were manually confirmed by an experienced laboratory technologist. A platelet cell count was also performed on the PRPr.

Serum and synovial SAA and TP concentrations were analysed as a batch using an automated chemistry analyser (Cobas Integra 400 Plus, Roche, Basel, Switzerland). Serum amyloid A concentrations were measured using an automated latex agglutination turbidimetric immunoassay (Eiken SAA, Eiken Chemical Company, Tokyo, Japan), previously validated for use in horses (Jacobsen et al. 2006a; Sanchez Teran et al. 2012). A nucleated cell count (NCC) was determined on the synovial fluid using an automated analyser (Cell Dyne 3700, Abbot Diagnostics, IL, United States). A direct smear for cytologic evaluation was prepared, after which the remaining sample was centrifuged for 8 minutes at 1520 g. A smear was made of the sediment, and the supernatant was stored at -80 °C. An experienced clinical pathologist who was blinded from the group distribution evaluated a direct and concentrated smear of the synovial fluid. A differential cell count was performed on all the fluid samples by microscopy.

Synovial fluid samples for growth factor analysis of PDFG-BB and TGF-*β*1 on Days 1 and 5 (post PRPr treatment) were transported on ice after collection. The samples were centrifuged at 1000 g for 15 min at 4 °C within 30 min of collection, and again at 10 000 g for 10 min at 2 °C – 8 °C within 2 h of collection to ensure complete platelet removal. The supernatant was stored at -80 °C for PDGF-BB and TGF-*β*1 concentrations to be determined as a batch. Growth factor concentrations on the synovial fluid were determined using a sandwich enzyme-linked immunoassay technique validated for use in horses (Human PDGF-BB and Human TGF-*β*1; Quantikine, R&D Systems) (Textor & Tablin 2013; Textor et al. 2013).

### Statistical analysis

Descriptive and comparative statistical analyses were performed using Stata 14 statistical software (StataCorp). The data were assessed for normality using the Shapiro–Wilk test. Linear mixed models were used to compare the concentrations of the blood and synovial fluid variables, as well as the synovial effusion and flexion score between the OA and control group on each day, within each group compared to Day 0 and for comparison of growth factor concentrations between Days 1 and 5. Bonferroni adjustment for multiple comparisons was used, and statistical significance was set at *p* < 0.05.

### Ethical considerations

The experimental protocol was approved by the University of Pretoria, Animal Ethics Committee (V011-15). Animals used were part of research herds belonging to the University of Pretoria.

## Results

### Platelet-rich product

Mean ± SD platelet concentration of the PRPr was 621 ± 200 ×10^9^/L (range: 396–1089), which was a 4.7-fold increase compared to the blood, 130 ± 26 ×10^9^/L (range: 99–171). The mean ± SD PRPr WBC count was 18.7 ± 4.5 ×10^9^/L (range: 12.9–26.1), which was a 2.1-fold increase compared to the blood, 8.9 ± 2.0 ×10^9^/L (range: 5.9–12.7).

### Clinical evaluation of joints

Variables are presented in Table 1. The mean ± SD OA score for the OA group was 1.80 ± 0.83. The synovial effusion score was significantly different between the control and OA group on Day 0 (*p* < 0.05) with a higher score in the OA group. Within the control group, the synovial effusion score was significantly elevated on Days 1 and 2 compared to Day 0 (*p* < 0.05). Although the mean synovial effusion score for the OA group was lower on Day 56 compared to Day 0, it was not significant. No significant differences were seen for flexion score. The statistical analysis of periarticular signs was not performed because of data type.

**TABLE 1:** Summary of clinical assessments for the control and osteoarthritis group (mean ± SD).

Group	Control	OA
**OA scale (0–3)**	0 ± 0	1.80 ± 0.83
**Synovial effusion score (0–3)**		
Day 0	0.00 ± 0.00**	1.00 ± 1.22**
Day 1	1.00 ± 0.00*	1.40 ± 0.89
Day 2	1.00 ± 0.00*	1.60 ± 0.54
Day 5	0.60 ± 0.54	0.80 ± 0.83
Day 21	0.60 ± 0.54	1.00 ± 0.70
Day 56	0.00 ± 0.00	0.40 ± 0.89
**Flexion score (0–3)**		
Day 0	0.00 ± 0.00	0.20 ± 0.44
Day 1	0.40 ± 0.54	0.40 ± 0.54
Day 2	0.00 ± 0.00	0.20 ± 0.44
Day 5	0.00 ± 0.00	0.20 ± 0.44
Day 21	0.00 ± 0.00	0.00 ± 0.00
Day 56	0.00 ± 0.00	0.00 ± 0.00
**Periarticular signs (yes/no)**		
Day 0	0/5	1/4
Day 1	5/0	3/2
Day 2	5/0	5/0
Day 5	3/2	2/3
Day 21	3/2	2/3
Day 56	0/5	0/5

OA, Osteoarthritis.

* , Statistically significant differences from baseline, within a given group (*p* < 0.05).

** , Statistically significant differences between groups on a specific day (*p* < 0.05).

### Synovial fluid cytology

**FIGURE 1:**
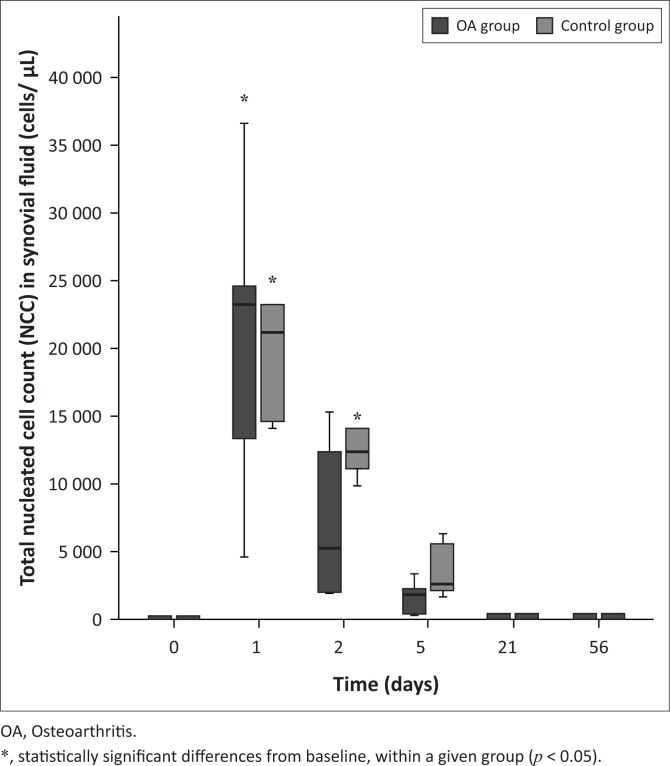
Total nucleated cell count in synovial fluid pre- and post-intra-articular platelet-rich product injection. On Day 1, both groups were significantly different from their corresponding baseline value. On Day 2, only control group was significantly different from baseline value.

Variables are presented in Table 2. The synovial NCC was significantly increased on Days 1 and 2 in the control group (*p* < 0.001 for both) and Day 1 in the OA group (*p* < 0.001) compared to Day 0 (see Figure 1). No significant differences were found between groups for Days 1 or 2. The NCC consisted predominantly of mature intact neutrophils. The neutrophil count was significantly increased on Days 1 and 2 in the control (*p* < 0.001 for both days) and OA (*p* < 0.001 for both days) groups compared to Day 0 but returned to normal numbers by Day 21. The neutrophil count was also found to be significantly higher in the control group on Day 2 compared to the OA group (*p* < 0.001).

**TABLE 2:** Summary of synovial fluid cytology, clinical chemistry and growth factor concentrations for control and osteoarthritis group (mean ±SD).

Day	NCC (cells/*μ*L)	Neutrophils (%)	TP (g/L)	SAA (mg/L)	PDGF-BB (pg/mL)	TGF-*β*1 (pg/mL)
**Day 0**						
Control	111.60 ± 39.89	1.60 ± 2.45	15.48 ± 6.31	0.00 ± 0.00	-	-
OA	87.40 ± 53.64	5.40 ± 8.50	14.22 ± 2.56	0.00 ± 0.00	-	-
**Day 1**						
Control	22940.00 ± 11103.28*^,^***	83.40 ± 2.70*^,^****	46.64 ± 11.11*	0.00 ± 0.00	390.47 ± 82.15**^,^***	4021.27 ± 783.00**^,^***,****
OA	20464.00 ± 12055.93*^,^***	71.20 ± 5.40*^,^****	39.06 ± 7.10*	0.00 ± 0.00	302.27 ± 47.44**	2792.48 ± 615.46**^,^***,****
**Day 2**						
Control	13212.00 ± 3413.46*	64.00 ± 4.00*^,^**	30.18 ± 11.55*	0.00 ± 0.00	-	-
OA	7372.00 ± 6122.10	38.80 ± 14.32*^,^**	22.46 ± 5.90*	0.00 ± 0.00	-	-
**Day 5**						
Control	3654.00 ± 2037.59***	14.80 ± 11.86****	20.56 ± 8.02	0.95 ± 2.14	316.42 ± 45.69***	851.73 ± 351.82***,****
OA	1660.40 ± 1250.72***	8.20 ± 15.54****	16.54 ± 6.09	0.00 ± 0.00	318.06 ± 37.82	678.35 ± 289.58***,****
**Day 21**						
Control	207.60 ± 49.90	2.60 ± 1.67	17.14 ± 6.61	0.00 ± 0.00	-	-
OA	213.00 ± 123.80	5.20 ± 6.97	12.18 ± 3.18	0.00 ± 0.00	-	-
**Day 56**						
Control	224.80 ± 71.94	10.80 ± 11.16	14.62 ± 4.51	0.00 ± 0.00	-	-
OA	95.60 ± 71.40	4.20 ± 6.79	13.78 ± 3.26	0.00 ± 0.00	-	-

OA, Osteoarthritis; NCC, nucleated cell count; TP, total protein; SAA, Serum amyloid A; PDGF, platelet-derived growth factor; TGF-*b*1, transforming growth factor-beta 1.

* , Statistically significant differences from baseline, within a given group (*p* < 0.05).

** , Statistically significant differences between groups on a specific day (*p* < 0.05).

*** , Statistically significant correlation between NCC (cells/*μ*L) and PDGF-BB/TGF-*b*1 (*p* < 0.05).

**** , Statistically significant correlation between Neutrophils (%) and PDGF-BB/TGF-*b*1 (*p* < 0.05).

### Synovial fluid growth factor concentrations

**FIGURE 2:**
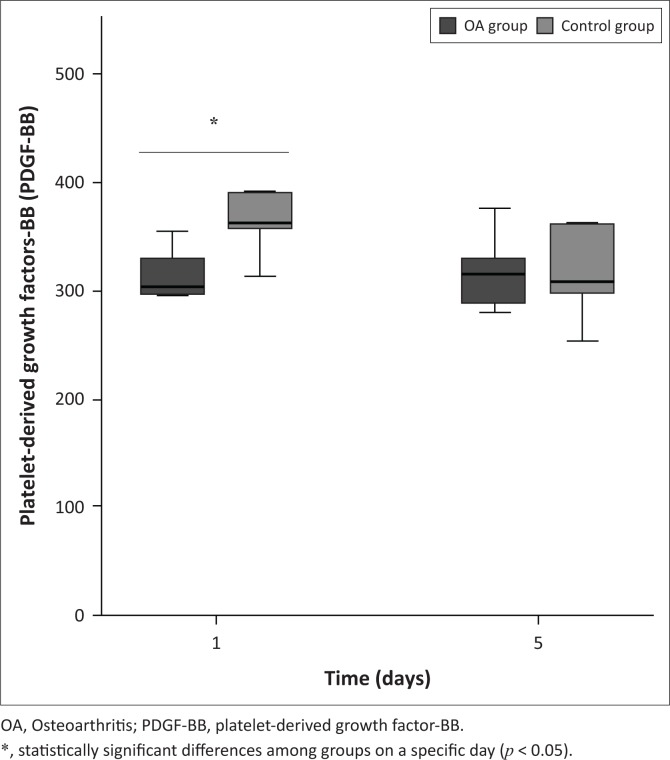
Mean synovial fluid platelet-derived growth factor-BB concentration after intra-articular platelet-rich product injection. Platelet-derived growth factor-BB was detected in both groups and was statistically significant among groups on Day 1.

**FIGURE 3:**
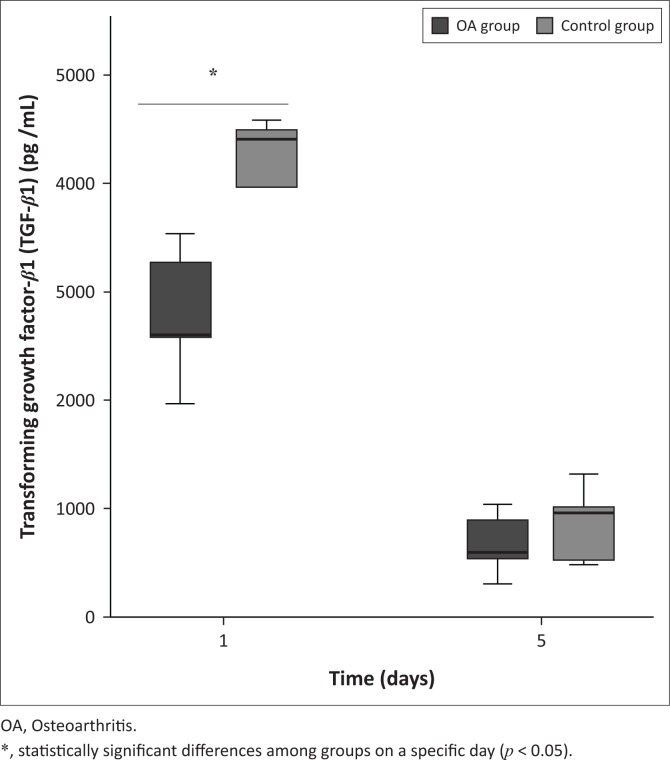
Mean synovial fluid transforming growth factor-beta 1 after intra-articular platelet-rich product injection. Transforming growth factor-beta 1 was detected in both groups and was statistically significant between groups on day 1.

Variables are presented in Table 2. The mean concentrations of PDGF-BB (Figure 2) and TGF-*β*1 (see Figure 3) were increased in both groups; however, concentrations were significantly lower in the OA group on Day 1 (*p* < 0.01 and *p* < 0.001, respectively) compared to the control group. On Day 5, the mean PDGF-BB concentration was unchanged in both the groups, compared to Day 1, with no significant difference between the groups. However, the mean TGF-*β*1 concentration decreased in both the groups on Day 5 compared to Day 1 with no significant difference between the groups. There was a significant correlation between TGF-*β*1 and NCC in both the control (*r* = 0.79; *p* < 0.05) and OA (*r* = 0.83; *p* < 0.05) groups, but PDGF-BB only showed a significant correlation with NCC in the control group (*r* = 0.80; *p* < 0.05). Only TGF-*β*1 showed a significant correlation with the synovial neutrophil percentage in both the control (*r* = 0.94; *p* < 0.05) and OA (*r* = 0.94; *p* < 0.05) groups (Table 2).

### Synovial fluid and serum total protein and serum amyloid A concentrations

The mean synovial fluid TP concentration was not significantly different between groups on Day 0. There was a significant increase in the mean synovial fluid TP concentration on Days 1 and 2 for the control (*p* < 0.001 for both) and OA (*p* < 0.001 and *p* < 0.01, respectively) groups, compared to Day 0. The concentration returned to normal for all horses by Day 56. The serum and synovial fluid SAA concentrations remained below the low detection limit of quantification for both groups and were not compared statistically.

## Discussion

Intra-articular PRPr administration resulted in high concentrations of synovial growth factors, PDGF-BB and TGF-*β*1, as well as a transient synovial inflammatory reaction in both the groups and an increase in synovial effusion of the OA group.

The PRPr preparation system used in this study has been evaluated before and was found to be safe in the treatment of normal equine joints (Textor & Tablin 2013). Our study confirmed that the use of this PRPr preparation system was safe for use in OA equine joints. This system provides equine practitioners with a stable-side preparation and immediate administration of a PRPr of known composition and quality. The preparation and processing of PRPr in the study was rapid and self-reliant.

The joints selected are representative of joints with a high prevalence of OA in the equine industry (Mcllwraith 2016). To date, there is no standardised radiological OA grading system for horses, except for the brief classification used by Frisbie et al. (2007). Comprehensive classification systems exist for OA in humans, of which Kellgren–Lawrence grading system (Holzer et al. 2015) is an example. This system has been used for the classification of OA in dogs (Nganvongpanit et al. 2014).

The PRPr was not activated, as exogenous platelet activation is not commonly used in equine practice, and platelets can be activated upon exposure to synovial fluid. (Textor et al. 2013; Yin, Shanmugavelayudam & Rubenstein 2011). Platelet-rich plasma may also be activated using CaCl_2_, thrombin, chitosan or batroxobin (Textor & Tablin 2013). Side effects associated with thrombin as an activator for intra-articular-administered PRP include joint effusion, pain on flexion and periarticular heat. These effects are not seen during resting-PRPr and CaCl_2_-PRPr treated joints (Textor et al. 2013). It is recommended that bovine thrombin should not be used for the activation of PRP for intra-articular administration (Textor & Tablin 2013). The growth factor concentration analysis from activation techniques found that no external activation is required, as the growth factors are released spontaneously upon exposure to synovial fluid because of the shear forces experienced by platelets from the highly viscous fluid from healthy joints with similar growth factor concentrations achieved (Textor et al. 2013). Based on this finding, the PRPr for our study was not activated. Platelet activation upon exposure to synovial fluid is because of shear forces experienced by platelets from the highly viscous fluid (Textor et al. 2013; Yin et al. 2011). The physical interaction between hyaluronic acid in a synovial fluid and the latency-associated peptide (LAP) of growth factor TGF-*β* may be sufficient to break the non-covalent bonds linking the LAP to the growth factor, thereby releasing the active growth factor (Albro et al. 2012). As our results indicate lower growth factor concentrations in the OA group, this may be attributed to decreased synovial fluid viscosity in joints exhibiting OA and, thus, may affect normal platelet activation by shear forces of synovial fluid (Albro et al. 2012; Chen et al. 2012; Conrozier et al. 2012; Textor et al. 2013). External platelet activation may be considered prior to intra-articular administration of PRPr in OA joints.

The significant clinical changes evident during our study were firstly, the higher synovial effusion score in the OA group compared to the control group on Day 0, which can be explained by the OA disease process. The control group synovial effusion score was significantly higher on Days 1 and 2 compared to Day 0, which may be attributed to the mild, transient but significant cellular inflammatory response present. This cellular inflammatory response found has also been reported in previous studies that investigated the effect of intra-articular injection of PRP or whole blood on the cytological composition of normal synovial fluid, which typically has a low cellularity (Judy & Galuppo 2005; Ovlisen et al., 2009; Textor & Tablin 2013). This finding may be attributed to cellular influx into the synovial fluid, serial arthrocentesis and leukocyte-rich PRPr composition used. The intra-articular administration of 15 million mesenchymal stem cells (autologous, allogeneic or xenogeneic) into normal fetlock joints of horses revealed a similar inflammatory response with a mean synovial NCC of greater than 40 000 cells/*µ*L and a mean TP greater than 40 g/L (Pigott et al. 2013).

Growth factors in this study were selected on the basis of their roles in the pathophysiology of OA (Fortier et al. 2011). Platelet-derived growth factor-BB and TGF-*β*1 have shown important anabolic/anti-inflammatory actions on joint synovial membrane and cartilage (Civinini et al. 2013). Both these growth factors lead to an increase in the production of extracellular matrix of cartilage, increased differentiation of synovial membrane cells in chondrocytes and a decrease in joint inflammation and pain (Ríos, López & Carmona 2015a).

Upon activation of PRP either by shear force from synovial fluid, direct contact with a constitutive collagen of treated tissue, calcium salts or thrombin, it forms what is known as platelet-rich gel (Giraldo, Álvarez & Carmona 2017). This acts as a scaffold that is capable of sequestering and releasing growth factors. Growth factors can be rapidly induced and detected within 1 h for TGF-*β* and 6 h for PDGF-BB (Textor et al. 2013). Thus, in this study, growth factor concentrations were determined on Days 1 and 5. For the limited number of studies that evaluated PRP growth factor release, growth factor concentrations were measured up to 96 h (Giraldo et al. 2017; Ríos et al. 2015b; Textor et al. 2013).

Under physiological conditions, active growth factor concentrations, such as PDGF-BB and TGF-*β* in joints are normally low or undetectable in healthy joints (Textor et al. 2013; Van der Kraan 2017). In cases with OA, high concentrations of active TGF-*β* have been reported (Van der Kraan 2017). Unfortunately, most studies including our study only reported the total synovial fluid TGF-*β* concentrations, which makes it difficult to make a statement about active TGF-*β* concentrations *in situ* (Van der Kraan 2017).

In our study, high concentrations of synovial PDGF-BB and TGF-*β*1 were present in both the control and OA horses on Day 1 after intra-articular administration of PRPr. These concentrations were, however, significantly lower in the OA group compared to the control group on Day 1. Studies have demonstrated the effect of age on TGF-*β* concentrations in synovial fluid and its effect on proteoglycan synthesis in cartilage (Iqbal et al. 2000; Wei & Messner 1998). Higher synovial fluid concentrations of TGF-*β* were found in younger animals compared to adults resulting in higher cartilage proteoglycan synthesis, which may contribute to the better healing capacity seen in younger animals (Iqbal et al. 2000; Wei & Messner 1998). Both Iqbal et al. (2000) and Nixon, Brower-Toland and Sandell (2000) reported that TGF-*β* expression in young equine adult cartilage is increased and declines with ageing of cartilage. The age differences between the two groups in our study might have contributed to the higher synovial growth factor concentrations in the control group compared to the OA group. Another possibility for the lower growth factor concentrations in the OA group may be attributed to the decreased synovial fluid viscosity in joints exhibiting OA, which may have affected normal platelet activation and growth factor release (Albro et al. 2012; Chen et al. 2012; Conrozier et al. 2012; Textor et al. 2013).

An important factor that should be considered in platelet concentration technologies is not the quantity of platelets, but rather how platelets, leukocytes, activating factors and growth factors interact in the final product. A strict quantitative approach does not define the biological signature and mechanism of action of the product, but rather the qualitative properties of the final product must be considered. Platelet-rich plasma classification systems for horses have recently been developed to assist with the standardisation of this therapy. Pure PRP, also known as leuco-reduced PRP, displays slightly higher platelet counts (1.3–4.0 fold) and leukocyte counts (0.5–2.0 fold) when compared to baseline whole blood, while leucocyte-rich PRP displays higher platelet counts (5-fold) and leukocyte counts (3-fold) when compared to baseline whole blood (Giraldo et al. 2017; Ríos et al. 2015a). Controversy still exists on which PRP system is best suited for horses. Evidence suggests that the leukocytes in leucocyte-rich PRP systems may enhance PRP growth factor concentrations, either through release of growth factors from leukocytes within the PRP or by acting as a stimulus for the release of growth factors from platelets (Giraldo et al. 2017; Zimmerman, Jakubietz & Jakubietz 2001). Dohan Ehrenfest et al. (2012) also found a strong influence of the leukocyte populations in PRP on the release of growth factors, particularly TGF-*β*1. Platelet-derived growth factor-BB concentration of the PRP was also shown to be correlated with the platelet and leukocyte concentrations in the PRP (Roh et al. 2016). In our study, the PRPr can be classified as a leukocyte-rich PRP. A statistically significant correlation was found between synovial fluid NCC and TGF-*β*1 for both the control and OA groups, and PDGF-BB for the control group. Thus, the leukocyte-rich PRPr used may also contribute to the detected synovial fluid growth factor concentrations.

The persistently elevated PDGF-BB concentration in both the control and OA groups may be attributed to the slow, continuous release of PDGF-BB. This finding differs from previous reports that showed PDGF-BB to be decreased 6 h post intra-articular injection (Textor et al. 2013). In contrast, PDGF-BB release can be constant and sustained over 7 days (Roh et al. 2016). These results are also consistent with the findings reported by Mazzucco et al. (2009) where the individual dynamics of the growth factors released depended exclusively on the type of growth factor, rather than on the preparation method.

Serum amyloid A is a sensitive marker of joint disease (Jacobsen et al. 2006b). In this study, the serum and synovial concentrations of SAA in both groups remained undetectable despite the elicited inflammatory reaction. The inflammatory reaction may not have been severe enough to elicit elevated concentration of SAA. Within the OA group, undetectable SAA concentrations may be attributed to the chronicity of the condition. This finding was similar to a previous report where SAA concentrations were evaluated in serum and synovial fluid samples from healthy horses and horses with infectious arthritis and OA (Jacobsen et al. 2006b). The latter study found SAA to be a good marker of infectious arthritis cases, but OA cases had low concentrations of SAA in serum (0.48 mg/L) and synovial fluid (0.7 mg/L), suggesting decreased production of SAA because of the chronicity of the OA as it is synthesised only in response to moderate to severe active inflammation. In addition, repeated arthrocentesis also was shown not to cause a detectable increase in serum and synovial SAA (Jacobsen et al. 2006b; Sanchez et al. 2008). The increased synovial TP concentration in our study, after intra-articular administration of PRPr, has been previously described and was ascribed to a mild acute inflammatory response elicited by intra-articular administration of PRPr, as well as repeated arthrocentesis (Jacobsen et al. 2006b; Sanchez Teran et al. 2012).

The study had several limitations. Firstly, the control group was not age-matched to the OA group. This was as a result of specific joint selection that did not exhibit radiographic changes of OA for the control group. Osteoarthritis is a degenerative disease that is commonly seen in older aged horses; thus, a control group of the same age distribution would be improbable. The small sample size because of the constraints of limited funding was another limitation. The lameness evaluation performed during the study period would have added valuable information to the study, but this was influenced by unforeseen external factors. Lastly, although the main aim of the study was to determine the response of an OA joint to intra-articular administration of a PRPr, a control OA group treated with intra-articular saline may have given additional information about the inflammatory reaction in OA joints for comparison.

On the basis of the results obtained in this study, it can be concluded that a stable-side PRPr preparation with known composition and quality can be administered rapidly and safely on horses. The external activation of PRPr may be advisable prior to its intra-articular administration.
